# No adverse effect of a maternal high carbohydrate diet on their offspring, in rainbow trout (*Oncorhynchus mykiss*)

**DOI:** 10.7717/peerj.12102

**Published:** 2021-09-08

**Authors:** Therese Callet, Hongyan Li, Anne Surget, Frederic Terrier, Franck Sandres, Anthony Lanuque, Stephane Panserat, Lucie Marandel

**Affiliations:** 1 Institut National de la Recherche pour l’Agriculture, l’Alimentation et l’Environnement (INRAE), Saint-Pée-sur-Nivelle, France; 2 State Key Laboratory of Freshwater Ecology and Biotechnology, Institute of Hydrobiology, Chinese Academy of Sciences, Wuhan, China; 3 University of Chinese Academy of Sciences, Beijing, China

**Keywords:** Aquaculture, Nutritional Programming, Salmonid, Plant-derived carbohydrates

## Abstract

In order to develop a sustainable salmonid aquaculture, it is essential to continue to reduce the use of the protein-rich fishmeal. One promising solution to do so is the use of plant-derived carbohydrates in diet destined to broodstock. However, in mammals, the reduction of protein content (replaced by carbohydrates) in parental diet is known to have strong adverse effects on offspring phenotypes and metabolism. For the first time, the effect of a paternal and a maternal high carbohydrate-low protein diet was assessed on progeny at long term in the rainbow trout. A 30% protein diminution in both males and females broodstock diet during 10 month and 5 months, respectively, did not trigger adverse consequences on their offspring. At the molecular level, offspring transcriptomes were not significantly altered, emphasizing no effect on metabolism. Tenuous differences in the biochemical composition of the liver and the viscera were observed. The recorded effects remained in the normal range of value and accordingly offspring growth were not negatively affected over the long term. Overall, we demonstrated here that a 30% protein diminution during gametogenesis is feasible, confirming the possibility to increase the proportion of plant-derived carbohydrates in female broodstock diets to replace fishmeal proteins.

## Introduction

The aquaculture industry is constantly developing better feeds: feeds that are both covering the fish known nutritional requirements and being economical and environmentally friendly. To this end, the research in aquaculture nutrition has notably focused on the replacement of fishmeal and fish oil, traditional ingredients used for rearing aquaculture carnivorous species (Naylor et al., 2009). Plant-derived carbohydrates appear to be good candidates to replace protein included in fishmeal, as they could represent a non-negligible source of energy, help sparing proteins for growth (Hemre, Mommsen & Krogdahl, 2002) and as they are more available in Europe and cheaper than fishmeal (Prabu et al., 2017). Such substitution will however lead to the decrease of the protein-to-carbohydrate ratio as fishmeal are rich in protein but devoid of carbohydrates (Jobling, 2012).

Even though broodstock have a high protein requirement but no specific requirement for dietary carbohydrates, a study on rainbow trout has recently demonstrated that female trout broodstock are able to grow and reproduce normally over an entire reproductive cycle when fed a diet in which fishmeal have been partially replaced by plant-derived carbohydrates (Callet et al., 2020). Thereby, such ingredients could be a viable solution to replace fishmeal in diets for broodstock.

Nevertheless, it is now recognized that nutritional insults occurred during the peri and prenatal life could affect an individual at long term. As such, both the maternal and the paternal diets could affect their offspring metabolism, phenotypes and health at long term (Rando & Simmons, 2015; Guo, Luo & Lin, 2020), in a gender specific manner (Tarrade et al., 2015). This concept is well known as programming or DOHAD (Developmental Origins of Health and Disease) in mammals (Hoffman, Reynolds & Hardy, 2017). In mammals, both the over/under nutritions and an altered macronutrients balance have been studied (Guo, Luo & Lin, 2020). Among the latter, protein restriction and increased carbohydrates proportion in parental diets are known to have deleterious effects on offspring such as the reduction of their lifespan, development of obesity, glucose intolerance and modification of cholesterol metabolism in mammals (Langley-Evans, 2009; Hoffman, Reynolds & Hardy, 2017; Guo, Luo & Lin, 2020). In teleost fish, the concept of programming has also been demonstrated (Hou & Fuiman, 2020). It could therefore be hypothesized that parental high carbohydrates (HC)/low protein (LP) diets could also affect the physiology and the metabolism of their offspring but such question has not been explored so far.

The present study aims to describe the consequences of a parental HC/LP diet in their offspring in carnivorous fish species. To do so, two-year old male and female rainbow trout, which is a commercially important salmonid species, were fed either a control diet (NC, 63.89% protein and 0% carbohydrates) or a “high carbohydrate/low protein” diet (HC/LP, 42.96% protein and 35.30% carbohydrates) for an entire reproductive cycle for females and for 5 months for males. Crossed-fertilizations were then carried out in order to obtain 4 groups of offspring (Callet et al., 2020). Zootechnical parameters were monitored until offspring reached a market fish size (portion trout). Moreover, transcriptomes of liver and muscle, which are two key tissues in metabolism and are known to be responsive to nutritional programming in trout (Song et al., 2018), were analysed to detect any impact on offspring metabolism.

## Materials and Methods

### Ethics approval

Investigations were conducted according to the guiding principles for the use and care of laboratory animals and in compliance with French and European regulations on animal welfare (Décret 2001-464, 29 May 2001 and Directive 2010/63/EU, respectively). This protocol and the project as a whole were approved by the French National Consultative Ethics Committee (reference numbers 201610061056842).

### Experimental design

Rainbow trout (*Oncorhynchus mykiss*) produced from broodstock with different nutritional histories were reared during 36 weeks to assess the effects of such nutritional histories.

**Figure 1:**
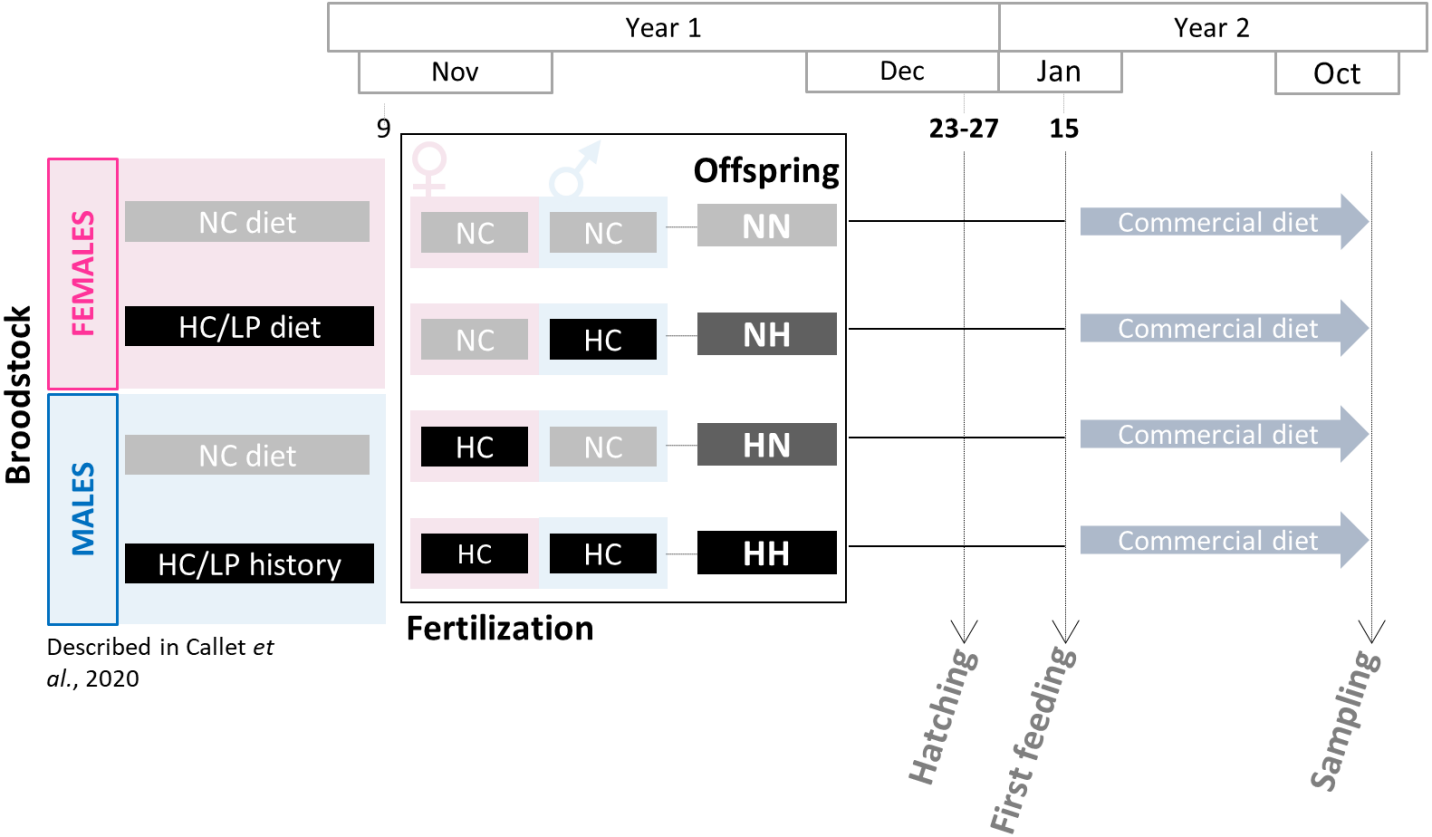
Experimental design. Broodstock females and males have been fed with either the NC diet (no-carbohydrates) or the HC/LP (high carbohydrates/low protein) diet. Cross-fertilizations were carried over to obtained four groups of offspring: NN, NH, HN and HH. Offspring were then fed during one year with a commercial diet to assess the effect of the parental HC/LP diet.

Broodstock feeding trial was previously described in Callet et al. (2020). Two-year old males and females rainbow trout were fed since the resumption of feeding with either a control diet (NC, 63.89% protein and 0% carbohydrates) or a low protein/high carbohydrate (HC/LP, 42.96% protein and 35.30% carbohydrates). The experiment has lasted 10 months for females and 5 months for males only as a *saprolegnia* infection has occurred (see details in Callet et al., 2020). During the spawning period (November-year 1), spawns from NC and HC/LP females were cross-fertilized with milts from males from each experimental condition in the experimental INRAE facilities of Lees-Athas (Fig. 1). Thus, offspring from 4 different conditions were obtained: the control NN offspring from both males and females fed the NC diet, HN offspring from only females fed the HC/LP diet and males fed the NC diet; NH offspring from only males fed the HC/LP diet and females fed the NC diet; and HH offspring from both parents fed the HC/LP diet. Eggs have hatched in December (year 1), from 44 to 48 days post-fertilization. Yolk-sac fry were then transferred to the INRAE experimental facilities of Donzacq (France). The offspring were reared into 12 tanks (n = 3/condition which is the number of tanks needed for nutritional studies (Jobling, 2012) in a flow-though reared system supplied with natural spring water at 17 °C. For each condition, from 1,300 to 1500 fish were randomly assigned to each of the three tanks. Tanks were randomly distributed in the experimental facilities and named without any indication of the offspring’s condition. Fish were examined daily and dead fish were removed. Since their first feeding (January-year 2), fish were fed *ad libitum* with a commercial diet (T3-P Omega, Skretting, France) during 36 weeks (until October-year 2). The initial density was adjusted after 3 weeks (150 fish per tank), 18 weeks (100 fish per tank), 21 weeks (70 fish per tank) and finally after 24 weeks (30 fish per tank). Between the 18 and the 21 weeks (mid-June-year 2), due to a technical issues, the water supply has been compromised in one NH tank, triggering a high mortality in this tank. To avoid any effect of the density, the fish from the 3 tanks were mixed and redistributed at equal density in the 3 tanks. Fish were then closely monitored and no consequences were detected after this event. During the growth trial, all fish were treated to prevent *flavobacterium* infections between 9–12 weeks and between 21–24 weeks (in April and June of year 2). Finally, a one day peak in deaths was recorded during the 27–30 weeks period, probably caused by an important stress due to a storm.

### Samplings

Every three weeks, fish were weighted and zootechnical parameters were calculated as follows (by tanks, *n* = 3 tanks):

Survival (%) = (N_*final*_/N_*init*_) ×100;

Specfic Gowth Rate (SGR,%/day) = 100 ×(ln(BW_*final*_)-ln(BW_*init*_))/day.

Dry feed intake was estimated by removing unconsumed (collected every day) from feed supplied. Feed efficiency (FE) and feed intakes were estimated as follows:

Feed efficiency (FE) = (BW_*final*_+BW_*d*_–BW_*init*_)/dry feed intake

Feed intake (FI) = 100 × dry feed intake/((BW_*final*_+BW_*init*_)/2)/day

Feed intake (FI_*MBW*_) = dry feed intake/((BW_*final*_^0.8^ × BW_*init*_^0.8^)^0.5^ × day)

with N_*init*_ and N_*final*_ the initial and final fish number; BW_*init*_, BW_*final*_ and BW_*d*_ the mass for initial, final and dead fish.

After 24 weeks and 36 weeks of feeding (July and October-year 2), fish were sampled. Fish were anesthetized with benzocaine (30 mg/L) and killed in a benzocaine bath at 60 mg/L. Concerning the 24-weeks-sampling, 9 fish from each condition were randomly sampled and stored at a whole at −20 °C for biochemical composition analysis. Concerning the 36-weeks-sampling (October-year 2), 9 individuals by condition were randomly sampled 6 h after the last feeding. Individual mean body weight (BW) and individual body length (L) were measured and the Fulton’s condition factor was calculated as K = 100*BW/L^3^ (*n* = 9). Blood was removed from the caudal vein and centrifuged at 3,000 g for 5 min. The plasma obtained was stored at −20 °C until further analysis. The viscera and hepatopancreas were removed and weighted. The viscerosomatic index (VSI) and hepatosomatic index (HSI) were calculated as HSI(%) = 100 × liver weight/fish weight and VSI(%) = 100 × viscera weight/fish weight (*n* = 9). The viscera, a part of the liver and a part of muscle were stored at −20 °C until biochemical analyses. Another part of the liver and muscle were removed and immediately frozen in liquid and stored at −80 °C until RNA extraction. Finally, the caudal fin of each fish were dissected and stored at −20 °C until DNA extraction.

### Biochemical composition

Proximate compositions of the feeds, whole-body fish samples (24-weeks-sampling), carcass, liver, muscle, and viscera (36-weeks-sampling) were analysed as follows: dry matter was determined by the oven drying to constant weight at 105 °C; ash was determined *via* combustion in a muffle furnace at 600 °C to a constant weight; crude protein (N ×6.25) was determined by the Kjeldahl method after acid digestion. Crude lipid in the diets and whole-body were determined by petroleum ether extraction (Soxthern), while total lipid content in the liver and muscle were performed using the dichloromethane/methanol (2:1, v/v) as the extraction liquid, according to Folch, Lees & Stanley (1957).

Hepatic glycogen and glucose content were performed in lyophilized liver. Glycogen content was determined following to the hydrolysis methods described by Good, Kramer & Somogyi (1933). Samples were ground in HCl (1 mol/L) and aliquot were made for two separate parts. For the free glucose measurement, one of the aliquot samples were detected using the Amplite Fluorimetric Glucose Quantitation Kit (AAT Bioquest, Inc., USA) after centrifuged at 10,000 g for 10 min. For the glycogen measurement, the other part of the ground tissue was boiled at 100 °C for 2.5 h, adjusted to neutralization by KOH (5 mol/L, VWR, USA), and determined using the same kits as above according to the manufacturer’s instructions. Finally, glycogen content was evaluated by subtracting free glucose content. Between 24 and 36 weeks, protein and lipid retentions (PRE and LRE) were also estimated as follows (*n* = 3):

PRE, LRE = (BW_*final*_ × X_*final*_ - BW _*init* *l*_ × X_*init*_)/NI_*x*_

with X_*init*_ and X_*final*_ the initial and final carcass content in protein/lipids (in g) and NI_*x*_ the protein/lipids intake (in g DM).

### Plasma metabolites

Plasma glucose, triglycerides, free fatty acids (FFA) and cholesterol concentrations were analysed with 4 kits (Glucose RTU, PAP 150 bioMerieux, Marcy l’Etoile, France, Fujifilm Wako, Sobioda and CHOD-PAP, Sobioda), according to the recommendations of manufacturer (*n* = 9).

### DNA extraction and determination of fry sex

DNA was extracted from the caudal fin of fish sampled after 36 weeks of feeding, using Chelex-100 (Bio-Rad Laboratories, CA, USA), following manufacturer’s instructions (*n* = 9). To assess offspring sex, the master sex-determining gene, *sdY* genes, was amplified by PCR from the extracted, as described in Yano et al. (2013). One µl of DNA were mixed with 1.25 µl of each primer (10 mM, forward: CCCAGCACTGTTTTCTTGTCTCA; reverse: CTGTTGAAGAGCATCACAGGGTC), 1 µl dNTP mixture and 5 µl of 5xPCR Buffer (Promega) with 0.125 µl of Taq DNA Polymerase (Promega) in a total volume of 25 µL. Thermal cycling consisted of denaturation for 20 s at 94 °C followed by 35 cycles of 94 °C for 20 s, 59 °C for 20 s, and 72 °C for 20 s, with a final extension of 5 min at 72 °C. PCR products were electrophoresed on a 2% agarose gel to reveal the presence or absence of sdY.

### RNA extraction

Liver and white muscle samples after 36 weeks were homogenised in Trizol reagent (Invitrogen, Carlsbad, CA, USA) using the Precellys 24 (Bertin Technologies, Montigny-le-Bretonneux, France). The total RNA was then extracted according to the Trizol manufacturer’s instructions (*n* = 9). The concentration of extracted RNA was analysed using a spectrophotometer (Nanodrop ND1000, LabTech) by measuring absorbance at 260 nm and quality of RNAs was checked with Bioanalyzer (Agilent Technologies, Kista, Sweden).

### Microarrays, cDNA labelling and hybridisation

Transciptome profiles of liver and muscles were analysed with microarray technology (Agilent-based microarray platform rainbow trout specific with 8 ×60 K probes), as previously described (Callet et al., 2018). Briefly, six RNA samples were selected among the 9 per condition, thanks to their RIN number. RNA was amplified by a reverse transcription, using a polyDT T7 primer (denaturation step: 10 min at 65 °C, reaction step: 2 h at 40 °C, inactivation step: 5 min at 70 °C) and the obtained cRNA were labelled with Cy3-dye (2 h at 40 °C). A RNeasy kit(Qiagen) was used to remove excess dye. The level of dye incorporation was evaluated using a spectrophotometer (Nanodrop ND1000, LabTech) (Yield ≥ 0.825 g cRNA and specific activity ≥ 6 pmol of Cy3 per µg of cRNA). 600 ng of Cy3-cRNA was then fragmented with a specific buer (30 min at 60 °C). Cy3-cRNA were then manually hybridised on a sub-array (17 h at 65 °C in a microarray hybridisation oven). After washing, slides were scanned (Agilent DNA Microarray Scanner, Agilent Technologies, Massy, France, parameters: 3 µm and 20 bits). Data were then obtained with the Agilent Feature Extraction software (10.7.1.1) and are available in the GEO database (ID: GSE169003).

### Statistical analyses

All data were presented as means ± standard deviation and the statistical analyses were performed using the R Software (version 3.2.5) (R Core Team, 2013). The significance threshold *p*-value was set at 0.05. Zootechnical parameters (mean body weight, survival, SGR, FI, FE, LRE and PRE) obtained throughout the trial were analysed using a Kruskal-Wallis test to assess the effect of the parental HC/LP diet. In case of a significant effect of the parental HC/LP diet, a Bartlett post-hoc test was carried out in order to decipher which condition was significantly different from the control NN fish.

Data obtained from the last sampling after 36 weeks of feeding were analysed thanks to linear mixed-effects models, using the packages “lme4” from the R software. The maternal HC/LP diet, the paternal HC/LP diet and the interaction between these two variables were investigated on all the parameters measured. As a sexual dimorphism in programming have been described in mammals (Tarrade et al., 2015), the effect of sex of the fish and the interaction between the sex and the parental HC/LP were thus also investigated on all the parameters. The tank was treated as a random effect. The best model based was then chosen thanks to the Akaike Information Criterion (AIC). Diagnostics plots were created for each model to evaluate the model assumptions. In case of a significant interaction, a Tukey post-hoc test was carried out.

Data from the microarray analysis were transformed with a logarithmic transformation, scale normalised and analysed using the package Limma (Ritchie et al., 2015). In order to find the differentially expressed genes resulting from the maternal, paternal and both maternal and paternal HC/LP diet, transcriptomes of HN, NH and HH liver and muscles were successively compared with the transcriptomes of the control NN fish. For these three comparisons, Limma t-tests were performed, with a correction for multiple tests (*P*-value cut-off = 0.05 after a Benjamini–Hochberg correction), taking into account the sex of the fish.

## Results

### Zootechnical parameters during the growth trial

During the trial, zootechnical parameters were monitored (File S1) and were affected differently depending on the period (Table 1). During the 3 weeks after the first feeding, while HN offspring have displayed a significantly lower growth and a lower final body weights, the NH and HH offspring have displayed a significantly higher growth and higher final body weights. Between 18 and 21, a high mortality occurred in one NH tank due to a technical issue (see Material and methods). This issue and the associated mortality triggered a diminution of feed intake in this same tank. Except from this period with the technical issue (18–21 weeks), NH and HH offspring had a higher FI but a lower FE during the growth trial. Their growth and final body weights were however never affected, except after 24 weeks when NH offspring have exhibited a higher mean body weights than the NN control one. Finally, HN and HH offspring have exhibited a slightly higher survival rates than the NN control fish during the one day peak mortality which occurred during the storm (see Material and Methods).

**Table 1:** Zootechnical parameters during the trial. Data are presented as mean ± standard deviation and analyzed by a kruskal-wallis test. In case of significant effect of the parental history, results are presented in bold (*P*-values ≤0.05) and a post-hoc Dunnet test was performed. Significant differences in comparison to the control NN group are represented with stars.

**Index**	**Parental history**				**Statistical**
	**NN**	**NH**	**HN**	**HH**	**analyses**
**0–3 weeks**					
Initial BW (g)	0.08 ±0.00	0.08 ±0.00	0.08 ±0.00	0.08 ±0.00	*ns*
**Final BW (g)**	**0.25 ±0.00**	0.27 ±0.00^***^	0.24 ±0.00^***^	0.27 ±0.00^***^	**0.02**
Survival (%)	100.0 ±0.00	100.0 ±0.00	100.0 ±0.00	100.0 ±0.00	*ns*
**SGR (%/day)**	**6.60 ±0.00**	6.95 ±0.00^***^	6.40 ±0.00^***^	6.97 ±0.00^***^	**0.01**
Feed intake (g DM/g/d)	1.42 ±0.16	1.33 ±0.18	1.43 ±0.14	1.22 ±0.19	*ns*
Feed intake (g DM/g^0.8^/d)	4.76 ±0.51	4.61 ±0.62	4.81 ±0.46	4.22 ±0.61	*ns*
Feed efficency	4.26 ±0.48	4.74 ±0.65	4.11 ±0.39	5.22 ±0.81	*ns*
**3–18 weeks**					
Final BW (g)	24.87 ±0.13	25.86 ±2.40	24.39 ±1.02	23.92 ±1.02	*ns*
Survival (%)	96.67 ±1.15	84.67 ±19.63	98.44 ±0.38	96.89 ±1.54	*ns*
SGR (%/day)	4.36 ±0.00	4.34 ±0.09	4.37 ±0.04	4.26 ±0.04	*ns*
**Feed intake (g DM/g/d)**	**1.43 ±0.01**	**1.50 ±0.06** ^**.**^	**1.42 ±0.01**	**1.47 ±0.02**	**0.03**
Feed intake (g DM/g^0.8^/d)	22.87 ±0.07	21.88 ±1.26	22.98 ±0.44	22.53 ±0.09	*ns*
**Feed efficency**	**1.31 ±0.01**	1.27 ±0.02^*^	**1.32 ±0.01**	1.27 ±0.02^*^	**0.03**
**18–21 weeks**	** **	** **	** **	** **	** **
Final BW (g)	46.06 ±1.29	51.29 ±3.02	45.40 ±2.27	45.27 ±0.61	*0.08*
**Survival (%)**	**100.00 ±0.00**	68.91 ±1.13^**^	**100.00 ±0.00**	**100.00 ±0.00**	**0.01**
SGR (%/day)	3.23 ±0.10	3.39 ±0.13	3.23 ±0.07	3.17 ±0.02	*ns*
Feed intake (g DM/g/d)	2.96 ±0.07	2.91 ±0.10	2.91 ±0.10	3.09 ±0.05	*ns*
**Feed intake (g DM/g** ^**0.8**^ **/d)**	**15.80 ±0.42**	14.55 ±0.58^*^	**15.45 ±0.67**	**16.40 ±0.29**	**0.04**
Feed efficency	1.05 ±0.03	1.09 ±1.03	1.08 ±0.02	0.99 ±0.01	*ns*
**21–24 weeks**	** **	** **	** **	** **	** **
**Final BW (g)**	**80.57 ±2.06**	89.40 ±2.67^**^	**83.44 ±2.47**	**82.95 ±1.62**	**0.05**
Survival (%)	100.00 ±0.00	100.00 ±0.00	99.52 ±0.82	100.00 ±0.00	*ns*
SGR (%/day)	2.31 ±0.08	2.32 ±0.12	2.53 ±0.13	2.46 ±0.02	*0.09*
**Feed intake (g DM/g/d)**	**2.53 ±0.24**	**2.69 ±0.02**	**2.64 ±0.03**	**2.76 ±0.03**	**0.05**
Feed intake (g DM/g^0.8^/d)	14.02 ±0.37	15.09 ±0.23	14.73 ±0.26	15.40 ±0.16	*0.07*
Feed efficency	0.89 ±0.07	0.84 ±0.04	0.93 ±0.04	0.86 ±0.01	*ns*
**24–36 weeks**					
**Survival (%)**	**86.67 ±5.77**	**92.22 ±3.85**	98.89 ±1.92^**^	97.78 ±1.92^*^	**0.04**
SGR (%/day)	1.51 ±0.03	1.39 ±0.08	1.47 ±0.01	1.45 ±0.08	*ns*
Feed intake (g DM/g/d)	1.42 ±0.11	1.53 ±0.04	1.36 ±0.06	1.50 ±0.03	0.07
**Feed intake (g DM/g** ^**0.8**^ **/d)**	**9.21 ±0.57**	**9.90 ±0.12** ^**.**^	**9.16 ±0.31**	10.01 ±0.07^**^	**0.03**
**Feed efficency**	**0.98 ±0.09**	0.82 ±0.04^*^	**0.95 ±0.04**	**0.86 ±0.05** ^**.**^	**0.05**
Protein retention (%)	28.24 ±7.98	24.02 ±3.24	29.73 ±5.39	26.40 ±2.43	0.06
Lipid retention (%)	64.78 ±7.98	62.40 ±3.24	73.98 ±5.39	69.42 ±2.43	*ns*

**Notes.**

*** *P*-value ≤ 0.001.

** *P*-value ≤ 0.01.

* *P*-value ≤ 0.05.

### Zootechnical parameters at the end of the growth trial

**Figure 2:**
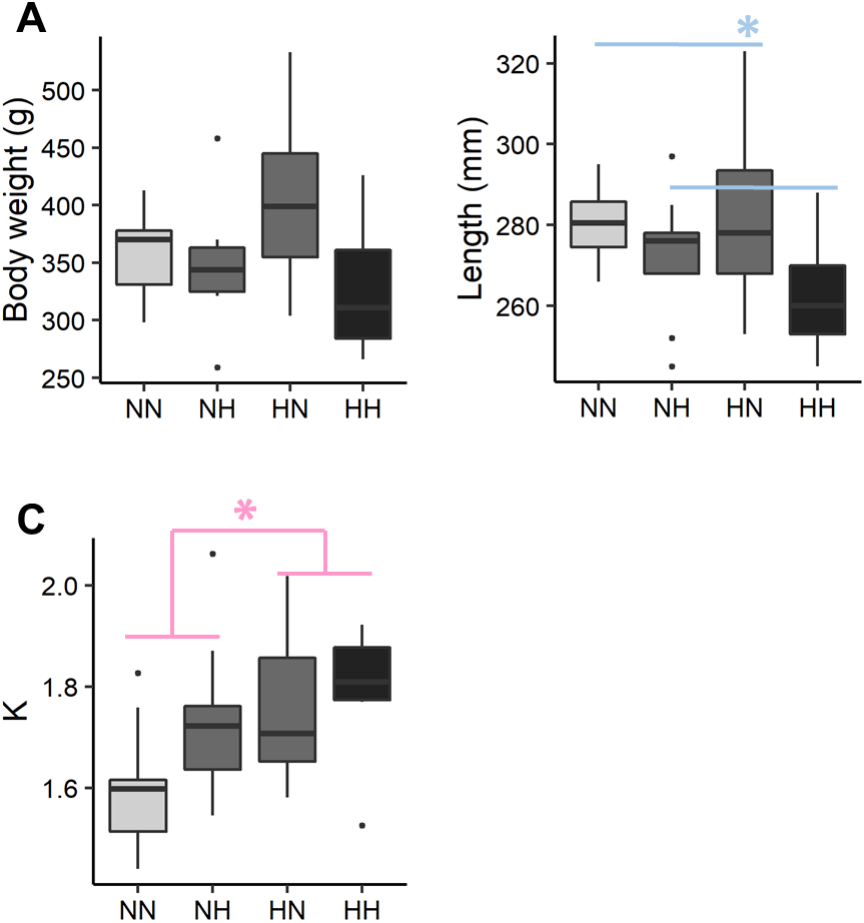
Zootechnical parameters. After 36 weeks of feeding, (A) individual body weights, (B) length and (C) the Fulton’s condition factor (K index) of the sampled fish (*n* = 9 per condition) have been analysed by a linear mixed-effects models. Significant differences due to the maternal HC/LP diet (red) and due to the paternal HC/LP (blue) are represented with stars (*P*-value < 0.05 ‘*’).

The individual body weight was not significantly different among the conditions (Fig. 2 and File S2 for the detailed statistical results). NH and HH offspring were significantly shorter than HN and NN ones regardless of their sex (−5.0%, *P*-value = 0.02). For HN and HH offspring, the Fulton’s condition factor was significantly increased in comparison to NH and NN ones, regardless of their sex (+6.5%, *P*-value = 0.04).

### Metabolic parameters at the end of the growth trial

**Figure 3:**
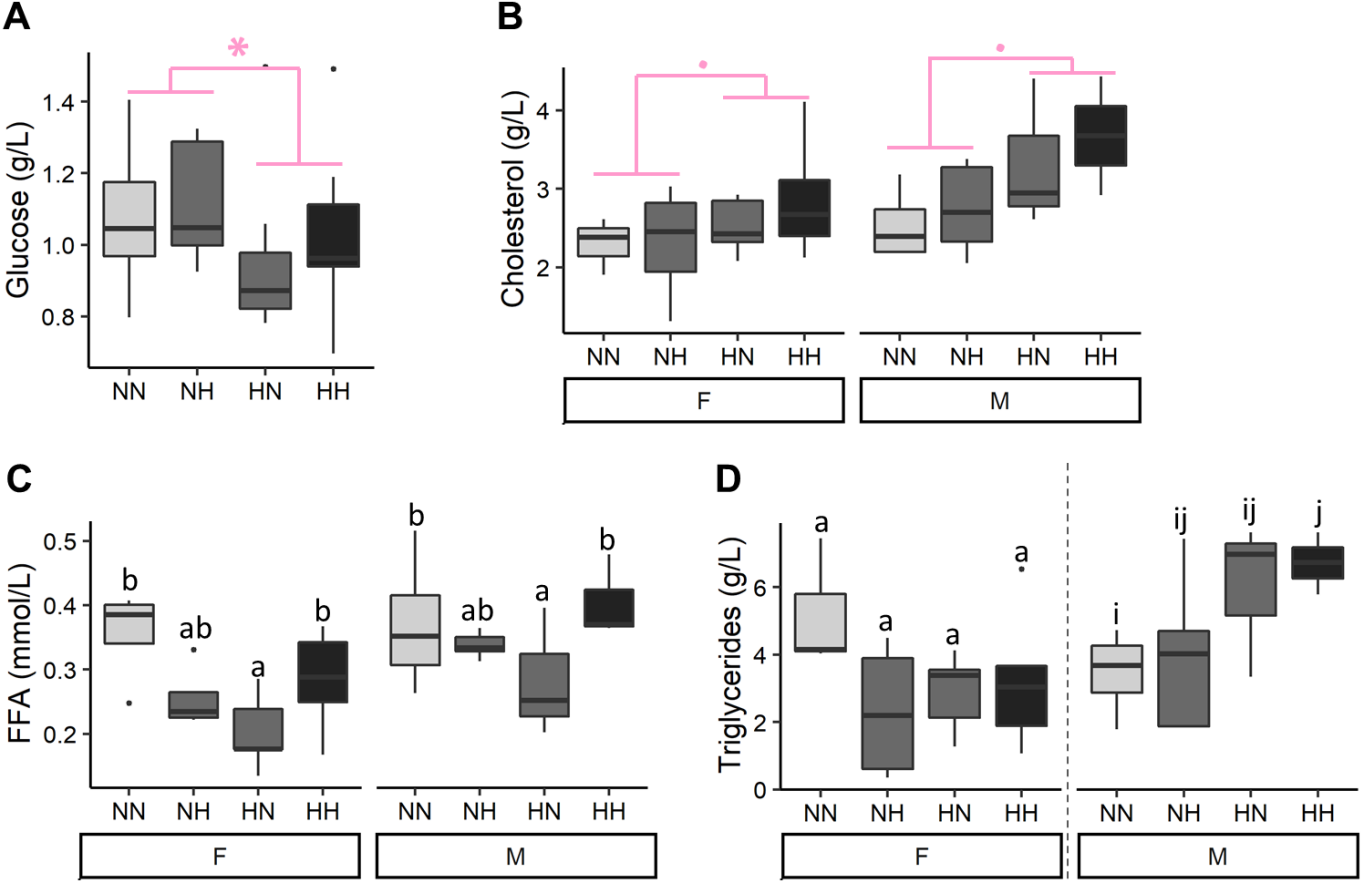
Plasma metabolites. (A) Glucose, (B) cholesterol, (C) free fatty acids (FFA) and (D) triglycerides levels in plasma of the sampled fish (*n* = 9 per condition) after 36 weeks of feeding. Except for plasma glucose concentrations, data are presented for offspring males (M) and females (F) as there was a significant effect of the offspring sex. Significant differences due to the maternal HC/LP diet are represented in red with stars (*P*-value < 0.10 ‘.’ and value <0.05 ‘*”). Different letters indicate significant differences between groups, which were investigated with a Tukey post-hoc test.

After 9 months of feeding, plasmatic parameters were measured (Fig. 3 and File S2 for the detailed statistical results). Offspring plasma glucose concentrations were significantly decreased in HN and HH offspring in comparison to NN and NH ones (−12.1%, *P*-value = 0.04). Females offspring had a significantly lower cholesterolemia than males ones (−13.3%, *P*-value = 0.009) and HH and HN offspring tended to have a higher cholesterolemia than the NH and NN ones regardless of their sex (+17.5%, *P*-value = 0.06). Females offspring had also a significantly lower FFA plasmatic level than males ones (*P*-value = 0.002) and the HN offspring had a 36.0% lower FFA plasmatic level than the control NN fish, regardless of their sex (*P*-value = 0.007). Finally, while no differences were detected in plasma triglycerides concentrations of females offspring, males HH had a significantly higher plasmatic triglycerides concentrations than NN ones (*P*-value = 0.02).

**Figure 4:**
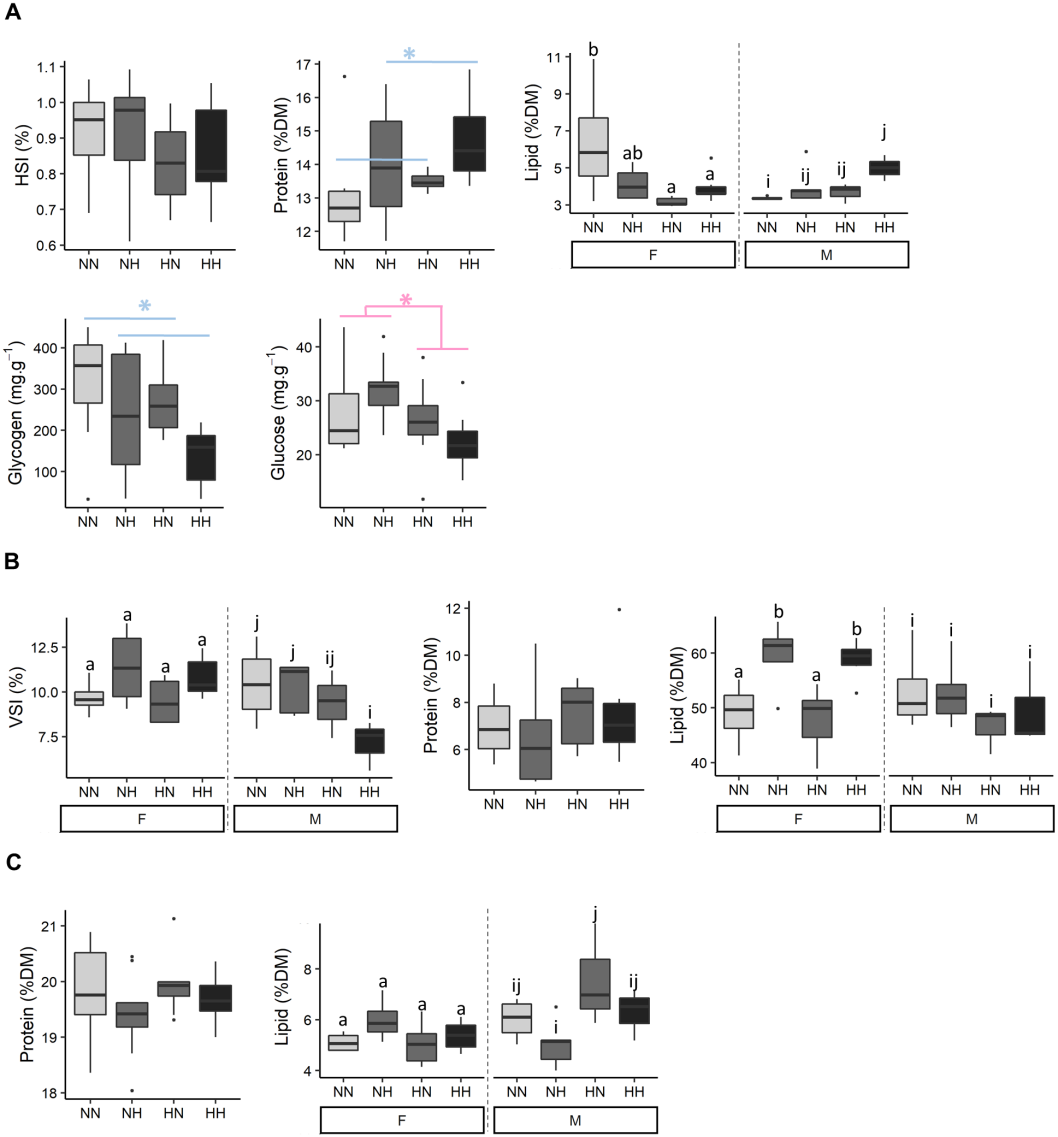
Tissue biochemical composition. The biochemical composition of (A) liver, (B) viscera and (C) muscle of the sampled fish (*n* = 9 per condition) after 36 weeks of feeding. Data are presented for offspring males (M) and females (F) when there was a significant effect of the offspring sex. Significant differences due to the maternal HC/LP diet (red) and due to the paternal history (blue) are represented with stars (*P*-value < 0.05 ‘*”). Significant interactions were investigated with a Tukey post-hoc test (significant differences are indicated with different letters).

Biochemical compositions of livers, viscus and muscles were analysed (Fig. 4 and File S2 for the detailed statistical results). No differences on HSI were detected among conditions. NH and HH offspring had a 8.6% higher protein content than the NN and HN ones (*P*-value = 0.04). While, females HN an HH had a lower hepatic lipid content than NN ones, males HH offspring had a higher lipid content than NN ones (*P*-value = 0.003). Also, NH and HH offspring had a 36.0% lower glycogen content than the NN and HN fish (*P*-value = 0.02). Finally, HN and HH offspring had a 19.6 % lower glucose content that NH and NN fish (*P*-value = 0.02).

While no differences on VSI were detected among females offspring, males HH offspring had a lower VSI than NN ones (*P*-value = 0.06). No significant differences were detected in the viscus protein content among offspring. Females NH and HH had a higher viscus lipid content than NN and HN ones (*P*-value = 0.003). No significant differences were detected in the muscle protein content among offspring. Muscle lipid content did not differ among females offspring, but males NH offspring had a significant lower lipid content than HN ones.

### Muscle and hepatic transcriptomes at the end of the growth trial

In muscle, 3 probes were found differentially expressed between HN and NN and 29 between HH and NN, irrespective of their sex (Table 2). Regardless of their sex, 18 probes were found differentially expressed between HH and NN offspring in liver (Table 3). No probes were found differentially expressed between NH and NN nor in liver, nor in muscle.

**Table 2:** Transcriptomic analyses in muscle. Probes differentially expressed between HN and NN; HH and NN in muscle, after limma t-tests (Adjusted *P*-value ≤0.05, Benjamini-Hotchberg).

**Description**	**LogFC**	**Adjusted**
		***P*-Value**
***HN vs NN***		
E3 ubiquitin-protein ligase HECTD2	−0.87	1.74E−02
Probable E3 ubiquitin-protein ligase HERC5	−1.27	1.92E−02
microtubule-associated protein 6 homolog	−0.73	3.39E−02
***HH vs NN***	** **	** **
Transposable element Tcb1 transposase putative mRNA	−1.37	3.35E−05
solute carrier family 2 member 6	−0.92	1.96E−02
*No description*	2.47	1.96E−02
60S ribosomal protein L22 putative mRNA	−1.13	2.03E−02
*No description*	1.06	2.03E−02
*No description*	−1.48	2.49E−02
dynamin-1-like protein	−0.84	2.49E−02
transcription factor PU.1-like	−0.82	2.49E−02
probable E3 ubiquitin-protein ligase HECTD2	−0.74	2.49E−02
ATP-sensitive inward rectifier potassium channel 11-like	−0.85	2.57E−02
MHC class I antigen (Onmy-U41p) pseudogene	−1.20	2.90E−02
*No description*	−0.99	2.90E−02
pyruvate dehydrogenase (lipoamide) beta	0.71	2.90E−02
*No description*	2.24	3.35E−02
Poly(ADP-ribose) polymerase 1	−0.67	3.40E−02
*No description*	0.60	3.40E−02
inward rectifier potassium channel 16-like	0.75	3.40E−02
ATP-sensitive inward rectifier potassium channel 10-like	−1.08	3.62E−02
myoferlin-like	0.55	3.74E−02
*No description*	−1.23	3.88E−02
Heterogeneous nuclear ribonucleoprotein A0	−1.03	3.88E−02
dehydrodolichyl diphosphate synthase	−0.92	3.88E−02
non-syndromic hearing impairment protein 5-like	−0.88	3.88E−02
Spi-1/PU.1 transcription factor	−0.72	3.88E−02
Transmembrane protein 136	−0.71	3.88E−02
exosome component 4	−0.68	3.88E−02
*No description*	−0.47	3.88E−02
*No description*	0.67	4.64E−02
unconventional myosin-X-like	1.53	5.00E−02

**Table 3:** Transcriptomic analyses in liver. Probes differentially expressed between HH and NN in liver, after a limma t-tests (Adjusted *P*-value ≤0.05, Benjamini-Hotchberg).

**Descriptions**	**LogFC**	**Adjusted**
		***P*-value**
***HH vs NN***	** **	** **
proton-coupled folate transporter-like	1.36	3.55E−02
nebulin-like	1.12	3.55E−02
protein enabled homolog	0.90	3.55E−02
acidic fibroblast growth factor intracellular-binding	0.88	3.55E−02
protein B-like		
*No description*	1.89	4.14E−02
*No description*	1.82	4.14E−02
afadin-like	1.37	4.14E−02
protein FAM219B-like	1.18	4.14E−02
single-stranded DNA-binding protein 3	1.01	4.14E−02
kinesin-associated protein 3-like	0.83	4.14E−02
*No description*	−0.80	4.14E−02
major histocompatibility complex class I-related	-2.01	4.14E−02
gene protein-like		
cytochrome P450 2B4-like	−2.06	4.14E−02
neuromedin-U-like	0.86	4.18E−02
cationic amino acid transporter 4-like	1.70	4.71E−02
lysine-specific demethylase 5C-like	−0.63	4.73E−02

## Discussion

Plant-derived carbohydrates appear to be a possible solution to replace fishmeal proteins in broodstock diets with higher trophic levels to progress toward a sustainable aquaculture (Callet et al., 2020). As it has been demonstrated in numerous studies that both the maternal and the maternal nutrition could affect their offspring in different fish species (Hou & Fuiman, 2020), consequences of a parental HC/LP diet need to be assessed and more particularly when they reach a market fish size.

### The paternal history had only slightly affected their offspring tissue composition

It is increasingly acknowledged that paternal diets, such as paternal HC/LP diets, could affect their offspring phenotypes and health at long term (Rando, 2012; Watkins et al., 2020). In the present study, due to a *Saprolegnia* infection which occurred during gametogenesis, the surviving males used for the reproduction could have been selected (Callet et al., 2020). The effect of the paternal HC/LP diet is thus combined with the effect of a potential selection. It is therefore impossible to decipher if the changes observed are due to the selection of males or due to the HC/LP diet received during the first period of gametogenesis, and we will refer such effects as paternal history effects.

The paternal history (HC/LP diet and selection) positively affect fish body weight at the beginning of the trial. However, such positive effect seems to fade along time as fish growth were not affected anymore beyond 3 weeks and fish length was slightly reduced at the end of the trial. Such outcome are probably resulting from the balance between a higher feed intake and a lower feed efficiency, reflected by a tendency to have a lower protein retention (Refer to Table 1).

Beside, at the end of the trial, the increase in hepatic glycogen content, which remained within known values for salmonids (Vijayan & Moon, 1992), was probably linked to the protein content decrease. Such phenotypes alterations are similar of what have been observed in offspring derived from males fed HC/LP diets in other species. For instance, it has been also shown that a HC/LP diet have modulated protein and glycogen content in *drosophila melanogaster* (Matzkin et al., 2011; Matzkin et al., 2013). Moreover, adiposity was increased in viscera of female offspring. Interestingly, even though these changes were limited (Kolditz et al., 2008), they are in agreement with results obtained in mice in which paternal HC/LP diets have also triggered an increase in body adiposity (Watkins et al., 2018). In mammalian models, such effects on phenotypes could arise from a perturbation of hepatic metabolism, which gene expression are symptomatic of non-alcoholic fatty liver disease (Watkins et al., 2018).

In spite of these differences observed in tissues composition, no specific metabolic pathway seemed to be affected by the paternal history as no genes were found differentially expressed in NH fish and only 18 were found in HH, regardless of their sex. Moreover, those 18 genes were not directly linked to intermediary metabolism. Because of a *saprolegnia* injury, the male broodstock were fed only during 5 months and were then re-fed with the control NC diet until reproduction (Callet et al., 2020). In addition to the severity of the nutritional insults, the window when is applied the insult has an importance for the imprinting of the nutritional programming. However, in fish, this critical window has not been clearly defined and the dietary shift could explain the small changes observed in NH and HH fish phenotypes and accordingly the unaffected transcriptomes.

### Offspring condition factor had been improved by the maternal HC/LP diet

It is well recognized that maternal low protein diets could affect their offspring phenotypes, metabolism and health at long term. More particularly, numerous studies have also showed that glucose homeostasis could also be affected in offspring derived from parents fed a HC/LP diet (Langley, Browne & Jackson, 1994; Desai et al., 1995; Ozanne & Hales, 2002). Regarding this, in the present study, the maternal HC/LP diet have slightly affected plasmatic glucose concentrations and the hepatic free glucose content in offspring. These changes were however only subtle (−12.1% for the plasmatic concentrations and −19.6% the hepatic free glucose content) and remained in the normal range of value found in salmonids (Congleton & Wagner, 2006). Moreover, no changes were detected at a molecular level as no genes were found differentially expressed in the liver of HN offspring and only 18 were found in HH ones, regardless of their sex. The only gene related to glucose metabolism differentially expressed between HH and NN fish was the gene coding for the Glut6 transporter (*solute carrier family 2 member 6*), which was down-regulated in the muscle of HH fish in comparison to the control NN fish. While maternal HC/LP diets are known to affect Glut4 expression in skeletal muscle (Zheng, Rollet & Pan, 2012), the role of Glut6, transporter belonging to the class III glucose transporters, has not been investigated yet. Together, these results suggested that the HC/LP diet did not altered their offspring metabolism.

Also, except from a reduction of their offspring body weights during the 3 first weeks of the trial, the maternal HC/LP diet did not compromise their offspring phenotypes. This negative effect of nutritional programming was then lost when fish grew in physiological condition and are not subjected to stressful situations. Such fading events along time has also been previously described in sea bass (Zambonino-Infante et al., 2019). At long term, the parental diet even improved their offspring Fulton’s condition factor, which mirrors fish health (Kroon, Streten & Harries, 2017). This augmentation could be of particular interest for the aquaculture sector. Such results in rainbow trout highly differed from other studies in mammals in which the maternal protein restriction during pregnancies could highly prejudice offspring health at long term, inducing obesity (Ozanne & Hales, 2002; Zohdi et al., 2015). Even though it has been reported that the effects of programming are strikingly constant across species (Langley-Evans, 2009), the differences reported here could be explained by two main reasons. First, because they have different developmental processes, mechanisms behind the programming events may highly differ in mammals and fish species, leading thus to different consequences. Second, the reduction of protein content in the HC/LP diet have reached 30% but still met the known nutritional requirement for rainbow trout broodstock (Jobling, 2012).

### Existence of a synergistic effect of the paternal and maternal HC/LP diet

Interestingly, the HH offspring have exhibited extreme value in comparison to the control NN fish for several of the recorded phenotypical traits. First, HH offspring had the highest K index, suggesting an improved health. However, their metabolism seemed to have been affected as they have also displayed the highest cholesterolemia and the highest plasmatic triglycerides levels (HH males only). HH offspring had also the most altered hepatic biochemical composition (highest protein content combined with the lowest glycogen and glucose content). HH males had also the highest lipid hepatic content in comparison to NN ones. Finally, males HH offspring had the lowest VSI. Such phenotype differences were logically associated with molecular data. Even if the number of genes differentially expressed between these two conditions remained low, HH offspring have the most reshaped hepatic and muscle transcriptomes in comparison to the control NN ones.

Therefore, the effects triggered by the paternal and by the maternal HC/LP diet seem to have cumulated. Such synergistic effect of parental nutrition have not been deeply investigated so far. Previous studies in mammals have however demonstrated that the negative effects induced by maternal and paternal condition (obesity) or nutrition (high sugar diet) could accumulate in their offspring (McPherson et al., 2015; Finger et al., 2015; Ornellas et al., 2016; Ornellas et al., 2019). A better understanding of the mechanisms behind the imprinting of programming events due to the maternal and the paternal is needed to better apprehend their possible interactions.

In addition to the interaction between the paternal and the maternal nutrition, the design of studies on programming in fish should also take into account the sex of the progeny. Although this was not the major purpose of our study, we have shown that males HH offspring appeared to be more sensitive to programming than females ones, confirming results obtained in mammals (Tarrade et al., 2015). As fish were raised together in tanks, without distinguishing sex, such effect have only been tested at the final sampling point (36 weeks) and it is thus not possible to decipher if the offspring’s sex have affected other zootechnical parameters of interest for aquaculture, such as feed intake or feed efficiency. These preliminary results however indicate that fish gender might modulate the effect of programming *via* a parental HC/LP diet.

## Conclusion

Despite the important reduction of the protein content in the diet of broodstock during gametogenesis (from 63.89% to 42.96%), no adverse effects of the parental history were recorded in offspring growth at long term. These results are highly contrasting with results obtained in mammals where the diminution of protein content in parental diet has strong negative effect on their offspring. Differences observed in tissues composition were negligible and remained in the normal range of value, which is confirmed by the molecular data. Of particular interest, the maternal HC/LP diet had even slightly increased fish condition factor, suggesting an improvement of fish health. Further studies should be carried out to validate this positive result. All together, this first study on the the long-term effect of a parental HC/LP diet in teleost fish confirms that HC/LP diets are suitable for such carnivorous species, supporting thus the feasibility to increase the proportion of plant-derived carbohydrates in broodstock diets to replace fishmeal proteins.

## Supplemental Information

10.7717/peerj.12102/supp-1 Supplemental Information 1 Zootechnical parameters monitored during the entire trial

10.7717/peerj.12102/supp-2 Supplemental Information 2 Statistical results of the linear mixed-effects models, concerning parameters recorded after 36 weeks of feeding

10.7717/peerj.12102/supp-3 Supplemental Information 3 ARRIVE checklist

10.7717/peerj.12102/supp-4 Supplemental Information 4 MIAME checklist

## Additional Information and Declarations

### Data Availability

The following information was supplied regarding data availability: The raw data are available in the Supplementary File. The microarray data are available at NCBI GEO: GSE169003.
