# Supplementary material for: No adverse effect of a maternal high carbohydrate diet on their offspring, in rainbow trout (Oncorhynchus mykiss)
Source: PeerJ. 2021 Sep 8;9:e12102. doi: 10.7717/peerj.12102 (PMC8434805; doi:10.7717/peerj.12102)
Supplement: Supplemental Information 4 [file peerj-09-12102-s004.pdf]

MIAME Checklist

Part 1 Experiment description

“The minimal information required in this section includes the type of the experiment (such as normal-versus-diseased comparison, time course, dose response, and so on) and the experimental variables, including parameters or conditions tested (such as time, dose, genetic variation or response to a treatment or compound).”
“this section specifies the experimental relationships between the array and sample entities—that is, which samples and which arrays were used in each hybridization assay. Each of these will be assigned unique identifiers that are cross-referenced with the information provided in the following sections.”

Rainbow trout were produced from broodstock with different nutritional histories. The control NN offspring from both males and females fed the control diet, HN offspring from only females fed a high carbohydrate/low protein (HC/LP) diet and males fed the control diet; NH offspring from only males fed the HC/LP diet and females fed the control diet; and HH offspring from both parents fed the HC/LP diet. Fish were reared during 36 weeks. Since their first feeding, fish were fed ad libitum with a commercial diet (T3-P Omega, Skretting, France). Transcriptomic analyses were carried out on liver and muscle tissues (n=6). Three comparisons were performed: HH vs NN, HN vs NN, NH vs NN.
(lines 67-79)

Part 2 Array design.

“The aim of this section is to provide a systematic definition of all arrays used in the experiment, including the genes represented and their physical layout on the array.”
“The array-type definition includes information common to all arrays of a particular type (such as glass-slide spotted with PCR-amplified cDNA clones) as well as precise descriptions of the physical content of each element (spot or feature). This section consists of three parts: (i) a description of the array as a whole (such as platform type, provider and surface type); (ii) a description of each type of element or spot used (properties that are typically common to many elements, such as 'synthesized oligo-nucleotides' or 'PCR products from cDNA clones'); and (iii) a description of the specific properties of each element, such as the DNA sequence and, possibly, quality-control indicators.”

The Array technology used is an Agilent Technologies, namely Agilent-071626 60k_trout_phylo_genome, series: GPL24910. This array is a in situ oligonucleotide technology, with a 8 X 60 K size (lines 162-164)

Part 3 Samples

“The MIAME 'sample' concept represents the biological material (or biomaterial) for which the gene expression profile is being established. This section is divided into three parts which describe the source of the original sample (such as organism taxonomy and cell type) and any biological in vivo or in vitro treatments applied, the technical extraction of the nucleic acids, and their subsequent labeling.”

Liver and white muscle were sampled in 9 fish from the four offspring condition (see above). Samples were homogenised in Trizol reagent (Invitrogen, Carlsbad, CA, USA) using the Precellys 24 (Bertin Technologies, Montigny-le-Bretonneux, France). The total RNA was then extracted according to the Trizol manufacturer's instructions. The concentration of extracted RNA was analysed using a spectrophotometer (Nanodrop ND1000, LabTech) by measuring absorbance at 260 nm and quality of RNAs was checked with Bioanalyzer (Agilent Technologies, Kista, Sweden). For each condition, 6 RNA samples were selected among the

9, thanks to their RIN number. 150 ng of total RNA was first amplified by a reverse transcription, using a polyDT T7 primer (denaturation step: 10 min at 65°C, reaction step: 2 hour at 40°C, inactivation step: 5 min at 70°C). The obtained cRNA were then labelled with Cy3-dye (2hr at 40°C). Excess dye was removed using a RNeasy kit (Qiagen). The level of dye incorporation was evaluated using a spectrophotometer (Nanodrop ND1000, LabTech) (Yield < 0.825 µg cRNA and specific activity < 6 pmol of Cy3 per µg of cRNA).
(lines 154-160 and 165-169)

Part 4 Hybridizations

"This section defines the laboratory conditions under which the hybridizations were carried out. Other than a free-text description of the hybridization protocol, MIAME requires that a number of critical hybridization parameters are explicitly specified: choice of hybridization solution (such as salt and detergent concentrations), nature of the blocking agent, wash procedure, *quantity of labeled target used*, hybridization time, volume, temperature and descriptions of the hybridization instruments."

600 ng of Cy3-cRNA was then fragmented with a specific fragmentation buffer and a 10× Gene Expression Blocking Agent, during 30 minutes at 60°C. Cy3-cRNA were then manually hybridised on a sub-array during 17 h at 65°C in a microarray hybridisation oven, according to Agilent's recommendations.
(lines 169-171)

Part 5 Measurements

"Image data should be provided as raw scanner image files (such as TIFF), accompanied by scanning information that includes relevant scan parameters and laboratory protocols."

Slides were washed and scanned (Agilent DNA Microarray Scanner, Agilent Technologies, Massy, France) using the standard parameters for a gene expression 8x60K oligoarray (3 µm and 20 bits). Data were then obtained with the Agilent Feature Extraction software (10.7.1.1).
(lines 171-173)

For each experimental image, a microarray quantification matrix contains the complete image analysis output as directly generated by the image analysis software (normally provided as separate spreadsheet-type files). Note that for a given image this is a 2D matrix, where array elements (spots or features) constitute one dimension and quantification types (such as mean and median intensity, mean or median background intensity) are the second dimension.

Data from the microarray analysis were first transformed with a logarithmic transformation and then scale normalised, using the package Limma from the R software. Data were then filtered: control and probes which were not expressed were removed from the analyses.
(lines 192-193)

Finally, the gene expression matrix (summarized information) consists of sets of gene expression levels for each sample. If microarray quantification matrices can be considered spot/image centric, then the gene expression matrix is gene/sample centric. At this point, the expression values may have been normalized, consolidated and transformed in any number of ways by the submitter in order to present the data in a form amenable to scientific analysis. Rather than attempting to impose a standard for gene expression values, MIAME indicates preferred detailed specifications of all numerical calculations applied to unprocessed quantifications in (b) that have led to the data in (c). Experimenters are encouraged, though not required, to provide reliability indicators (such as s.d.) for each data point.

Output files and normalized data are available in the GEO database (ID: GSE169003)

Part 6 Normalization controls

“A typical microarray experiment involves a number of hybridization assays in which the data from multiple samples are analyzed to identify relative changes in expression levels, identify differentially expressed genes and, in many cases, discover classes of genes or samples having similar patterns of expression.”

In order to find the differentially expressed genes resulting from the maternal, paternal and both maternal and paternal HC/LP diet, transcriptomes of HN, NH and HH liver and muscles were successively compared with the transcriptomes of the control NN fish.
For these three comparisons, Limma t tests were performed, with a correction for multiple tests (P-value cut-off = 0.05 after a Benjamini-Hochberg correction). Fold changes and associated p-values obtained are available in Table 2 and Table 3.
(lines 193-197)
